# Enhanced Sensing Enabled by Multi-Resonant QBIC-EIT and SP-BIC in Pyramidal LiNbO_3_ Metasurfaces

**DOI:** 10.3390/s26092632

**Published:** 2026-04-24

**Authors:** Changqing Zhong, Wei Zou, Jiangtao Lei, Yun Shen, Jing Chen, Lujun Hong, Tianjing Guo

**Affiliations:** 1 Department of Physics, School of Physics and Material Science, Nanchang University, Nanchang 330031, China; 402200230064@email.ncu.edu.cn (C.Z.); 352200250005@email.ncu.edu.cn (W.Z.); shenyun@ncu.edu.cn (Y.S.); 2 Institute of Space Science and Technology, Nanchang University, Nanchang 330031, China; jiangtaolei@ncu.edu.cn; 3 School of Physics, Nankai University, Tianjin 300071, China

**Keywords:** bound states in the continuum (BIC), electromagnetically induced transparency (EIT), high Q-factor, refractive index (RI) sensing, lithium niobate (LN) metasurfaces

## Abstract

In optical sensing, electromagnetically induced transparency (EIT) and bound states in the continuum (BIC) substantially enhance light–matter interactions by leveraging high-Q resonances. This study theoretically demonstrates dual-resonance phenomena—namely, a quasi-symmetry-protected BIC (SP-BIC) and a quasi-BIC-induced EIT-like (QBIC-EIT) resonance—using a dielectric metasurface composed of pyramid-shaped lithium niobate nanoarrays operating in the near-infrared. The QBIC-EIT transmission window originates from the interference between surface lattice modes and toroidal dipole modes, triggered by symmetry breaking of the BIC state. Due to the absence of C_4v_ rotational symmetry in the pyramidal unit cells, the metasurface exhibits pronounced polarization-dependent responses: Under x-polarized incidence, a single quasi-SP-BIC resonance appears; under y-polarization, dual quasi-SP-BIC resonances along with a distinct QBIC-EIT resonance are observed. Both the high-Q quasi-SP-BIC resonance and the EIT-like window show strong sensitivity to changes in the ambient refractive index (RI). Specifically, the EIT-like window achieves a sensitivity of 404.9 nm/RIU, while the quasi-SP-BIC resonance delivers an exceptional sensitivity of 887.7 nm/RIU, confirming the metasurface’s performance as a high-sensitivity RI sensor. These findings establish a multi-band detection platform for advanced RI sensing and contribute to the development of high-performance metasurface-based optical sensors.

## 1. Introduction

The advancement of nanophotonics is significantly driven by leveraging optical resonances to achieve exceptionally high radiative quality factors (Q-factors) [1]. A key phenomenon in this domain is electromagnetically induced transparency (EIT) [2,3], a coherent linear optical effect [4] initially observed in laser-driven three-level atomic systems under cryogenic conditions [5,6,7]. EIT manifests as a narrow transparency window within an absorption spectrum, arising from quantum interference effects [8], and holds substantial promise for diverse photonic applications [9,10,11]. However, its practical implementation in atomic systems is hindered by stringent requirements, including cryogenic temperatures [12], coherent pumping [6], and high optical intensities [13]. Recently, the demonstration of EIT-like behavior in plasmonic metamaterials has provided a more accessible pathway [14,15]. These structures enable EIT realization using incoherent light at room temperature. Consequently, plasmonic EIT metamaterials have found applications in slow-light devices, active plasmonic switching, and biological/chemical sensing [16,17,18,19,20,21]. Nevertheless, their performance is fundamentally limited by high intrinsic ohmic losses and irreversible photothermal conversion, leading to significantly constrained Q-factors [18]. To circumvent the substantial ohmic losses inherent in metallic structures, all-dielectric metasurfaces have emerged as a highly promising alternative [22,23]. EIT effects realized in such dielectric platforms offer advantages for low-loss slow-light devices, optical storage, and nonlinear optics [24,25,26]. Crucially, they exhibit significant potential for advanced refractive index (RI) sensing, where both sensitivity and figure of merit (FOM) are critical performance metrics [27,28,29].

Concurrently, bound states in the continuum (BICs) have garnered considerable attention in optical metasurface design confinement at the nanoscale [30,31,32]. BICs can be classified as symmetry-protected BICs (SP-BICs) or accidental BICs, distinguished by the mechanism decoupling their eigenmodes from the radiative continuum [33,34,35]. SP-BICs arise from specific structural symmetries (e.g., rotational or reflection symmetry) and can be transformed into observable Quasi-BICs (QBICs) with finite, yet high, Q-factors by intentionally breaking this symmetry to introduce controlled coupling to radiation [36,37]. Accidental BICs, conversely, stem from destructive interference between radiative channels [38]. High-Q BIC metasurfaces hold great promise for the design of nonlocal meta-lenses, enabling highly efficient asymmetric wavefront shaping [39]. Furthermore, high-Q metasurfaces have found widespread applications in advanced terahertz devices, including terahertz biosensing and terahertz absorbers [40,41].

Historically, research has often focused separately on Fano resonances, QBIC resonances, and EIT phenomena [32,42,43]. However, synergies exist: EIT in photonic systems can be mimicked by coupling resonators or modes, typically a radiative bright mode and a subradiant dark mode. The bright mode couples directly to the incident light, while the dark mode is indirectly excited by the incident light through coupling effects—such as quantum coherence between atomic energy levels or near-field coupling in artificial structures. Destructive interference between the bright and dark modes induces the formation of a transparency window. QBICs, acting as exceptional dark modes, are ideally suited for opening EIT channels when coupled to a bright mode [44,45]. This coupling, mediated by symmetry breaking and interference, generates a pronounced transparency window. Furthermore, the inherently high Q-factor of the QBIC results in an extremely narrow resonance linewidth, significantly enhancing the EIT window’s contrast and the system’s sensitivity. This mechanism unlocks new avenues for designing high-performance photonic devices, including sensors and slow-light elements [19,25]. Crucially, integrating both QBIC resonance and the EIT effect within a single system promises enhanced functionalities, such as multi-band RI sensing and optical switching, beyond what is achievable with isolated resonances or EIT alone [46,47].

In this work, we propose a lithium niobate (LN) metasurface with tilted pyramid unit cells. Under y-polarized normal incidence, it simultaneously supports a SP-BIC resonance, manifested as a toroidal dipole (TD) resonance, and a pronounced EIT-like window in the 1500–1600 nm band. The EIT-like originates from a destructive interference between a super-radiant lattice mode (SLM) [48] and the high-Q QBIC mode derived from the symmetry-broken TD mode. The metasurface exhibits strong polarization-selective responses: only the SLM mode appears under x-polarization in 1500–1600 nm, while distinct SP-BIC modes arise in 1300–1400 nm (one mode for x-polarization, two modes for y-polarization). The EIT-related resonance achieves a RI sensitivity of 404.9 nm/RIU, whereas the QBIC resonance reaches 887.7 nm/RIU. This design integrates multi-band BIC resonances and high-Q EIT in a single tunable platform, enabling advanced optical sensors. Compared with previously reported EIT-like or QBIC metasurfaces [44], our design eliminates the need for complex multilayer architectures [47] while maintaining resonance wavelengths that are nearly invariant with respect to the asymmetry parameter. This platform integrates multi-band BIC resonances and high-Q EIT effects within a single tunable configuration, offering significant advantages for advanced optical sensing. The pronounced polarization sensitivity of these high-Q resonances further enables polarization-controlled optical switching, where distinct QBIC modes can be selectively activated or suppressed by tuning the incident polarization state [49]. Additionally, the frequency-dependent response of such metasurfaces can be extended to space–time-coded designs, allowing for more versatile electromagnetic wave manipulation [50].

## 2. Structural Design and Methods

**Figure 1:**
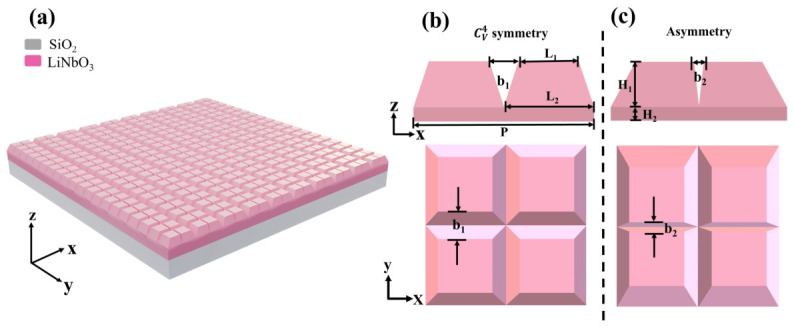
(**a**) Schematic of the multi-resonant metasurface featuring periodic LN pyramids. (**b**) Symmetric unit cell configuration with side and top views. (**c**) Asymmetric unit cell configuration with side and top views. The structure maintains a fixed period P = 1800 nm and has a pyramid geometry defined by top edge length L_1_ = 600 nm, bottom edge length L_2_ = 900 nm, and height H_1_ = 500 nm, supported by a substrate layer of thickness H_2_ = 140 nm. The thickness H_3_ of the silicon dioxide substrate is 500 nm.

We use a specific design of all-dielectric metasurfaces, which comprise four LN pyramids in each unit cell, as shown in Figure 1. The corresponding lattice constant is denoted by P = 1800 nm. Each pyramid stands at a height of H_1_ = 500 nm, with the lengths of its top and bottom edges measuring L_1_ = 600 nm and L_2_ = 900 nm, respectively. Supporting these pyramids is an LN plate, which has a thickness of H_2_ = 140 nm at the base of each pyramid. The substrate chosen for the metasurface is silicon dioxide with a refractive index of 1.4 and a thickness of 500 nm. This type of ultrathin silicon dioxide substrate can be grown on p-type (100) silicon wafers through thermal oxidation [51] or alternatively prepared using methods such as reactive magnetron sputtering and selective chemical etching [52]. The structure exhibits mirror symmetry in the z-y plane while incorporating intentional symmetry breaking in the z-x plane through the centerward offset of pyramids along the *x*-axis, as seen in Figure 1b,c, transitioning from intrinsic C_4v_ rotational symmetry (uniform inter-pyramid spacing b_1_ in both directions when untilted) to an asymmetric configuration that achieves quasi-SP-BIC states by reducing the x-directional slit spacing to b_2_, with the displacement parameter defined as b = (b_1_ − b_2_)/2. The LN pyramid metasurface structures can be fabricated through the following optimized procedure: a commercial LN film is first coated with a multilayer stack consisting of a thick resist, a chromium layer, and a second resist layer. The pattern is then defined using a variable-shaped beam electron writer, followed by sequential etching steps including reactive ion etching (RIE) for the chromium layer and reactive ion beam etching (RIBE) for the thick resist. Finally, the actual LN etching is performed via ion beam etching (IBE). This multi-step approach ensures both low sidewall roughness and high structural quality in the etched features [53,54]. The developed electric-field-driven nanoimprint technology enables the direct fabrication of large-area tilted metasurface nanostructures [55]. Numerous studies have utilized geometrically tilted structures to enhance the modulation capabilities of metasurfaces; for instance, tilted inverted pyramid structures have been employed to achieve efficient solar energy harvesting [56] and strong optical chirality [57]. Optical properties are simulated using COMSOL Multiphysics (v6.2) with Floquet–Bloch periodic boundary conditions applied to the x-z and y-z planes of the unit cell. The high Q-factors and pronounced field enhancement observed in BIC-based metasurfaces originate from collective modes sustained by their periodic lattice [58]. However, this inherent periodicity also renders the structures susceptible to finite sizes and edge effects. Since the formation of a well-defined quasi-BIC mode typically requires a minimum number of resonators (generally > 10 × 10) [59], the fabricated device dimensions must exceed this critical threshold to preserve the desired high-performance characteristics.

## 3. Excitation of Quais-SP-BIC and QBIC-EIT with Asymmetric Structures

**Figure 2:**
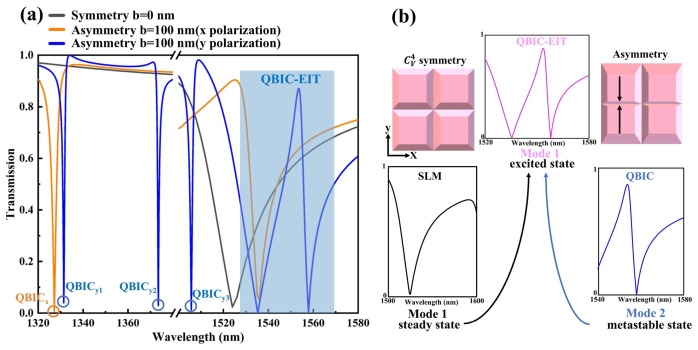
(**a**) The calculated transmission spectra for symmetric/asymmetric metasurface configurations under varied polarizations. The black line corresponds to the symmetric case. The orange line depicts x-polarized incidence on the asymmetric metasurface (b = 100 nm), marking the QBIC_x_ mode (orange circle). The blue line represents y-polarized incidence on the asymmetric structure, indicating QBIC_y1/2/3_ modes (blue circles). (**b**) Schematic of the formation of “QBIC-EIT” mode.

We investigate the proposed pyramid-shaped LN metasurface by calculating its transmission spectra under different polarization states over a wide wavelength range from 1320 nm to 1580 nm. The results are shown in Figure 2a. The range from 1380 nm to 1500 nm is omitted due to the absence of strong resonance features requiring a comparison between symmetric and asymmetric metasurface designs. When pyramid tilts are absent along the *x*-axis, the structure exhibits *C*_4v_ rotational symmetry, resulting in polarization-independent transmission, as presented by the black line in Figure 2a. It is clear that no resonance is observed within the 1320–1380 nm range, while a single broad-linewidth transmission dip appears in the 1500–1580 nm band, attributed to an SLM.

However, introducing a center-offset along the *x*-axis breaks the C_4v_ rotational symmetry, rendering the metasurface polarization-dependent, as evidenced by the orange and black lines in Figure 2a. Consequently, this symmetry breaking transforms the ideal SP-BIC into a QBIC state, thus generating a narrow-linewidth transmission resonance. For example, under x-polarized normal incidence, as presented by the orange line in Figure 2a, a distinct resonance emerges in the 1300–1380 nm band, characteristic of a quasi-SP-BIC. We designate this resonance as QBIC_x_ in Figure 2a. In contrast, the 1500–1580 nm band exhibits only the SLM, with no additional resonance features. Conversely, under y-polarized normal incidence, the asymmetric metasurface’s transmission spectrum, as presented by the blue line in Figure 2a, exhibits two distinct resonances within 1300–1380 nm. These modes—labeled QBIC_y1_ and QBIC_y2_ via blue circles—similarly constitute quasi-symmetry-protected BICs.

Furthermore, under y-polarized illumination, the metasurface’s transmission spectrum exhibits two additional resonances within the 1500–1580 nm band, which are at 1505 nm and 1555 nm, respectively, alongside the SLM. As shown by the blue line in Figure 2a, symmetry breaking induces a redshift in the SLM resonance. Critically, the QBIC mode at 1545 nm constructively interferes with the SLM, forming a novel “QBIC-EIT” hybrid mode [60]. This generation mechanism mirrors coherent interference in three-level atomic systems. Figure 2b shows the schematic diagram of the QBIC-EIT three-level Λ-type atomic system. In the photonic analogy of EIT, the system is modeled using Λ-shaped energy levels [10], comprising the ground state |1⟩, excited state |3⟩, and metastable state |2⟩. The transition from |1⟩ to |3⟩ is mediated by an SLM mode with a high decay rate, which can be directly excited by y-polarized incident light. The transition from |2⟩ to |3⟩ is characterized by a BIC mode. When the pyramid is not tilted, its radiative decay rate is nearly zero, preventing direct coupling with freely propagating waves in free space. To achieve an observable transition, the pyramid is tilted here to convert the ideal BIC into a measurable QBIC mode. When y-polarized light is normally incident on the asymmetric metasurface structure, quantum interference occurs. The transition amplitudes from Mode 1 to Mode 3 and Mode 2 to Mode 3 undergo destructive interference, creating a narrow transparency window in the spectrum, termed the “QBIC-EIT” mode. It should be noted that this hybrid mode is an EIT-like mode rather than Autler–Townes Splitting (ATS). This is because ATS arises from the frequency splitting of two Lorentzian peaks and does not involve destructive interference [61]. In contrast, the QBIC-EIT mode is generated by the destructive interference between the bright SLM mode and the dark QBIC mode. The coupling strength g between the SLM and QBIC modes is quantified via coupled-mode theory as g = Δω/2, with strong coupling occurring when $g>\left|\gamma_{SLM}-\gamma_{QBIC}\right|/2$ [44]. The coupling is primarily mediated by near-field overlap rather than the radiation continuum, as confirmed by spatial overlap integrals κ. Symmetry breaking (pyramid tilt) increases κ by a factor of 2–3, directly enhancing the coupling strength.

## 4. Results and Discussion

### 4.1. CMT Theoretical Fitting and Q-Factor Analysis

**Figure 3:**
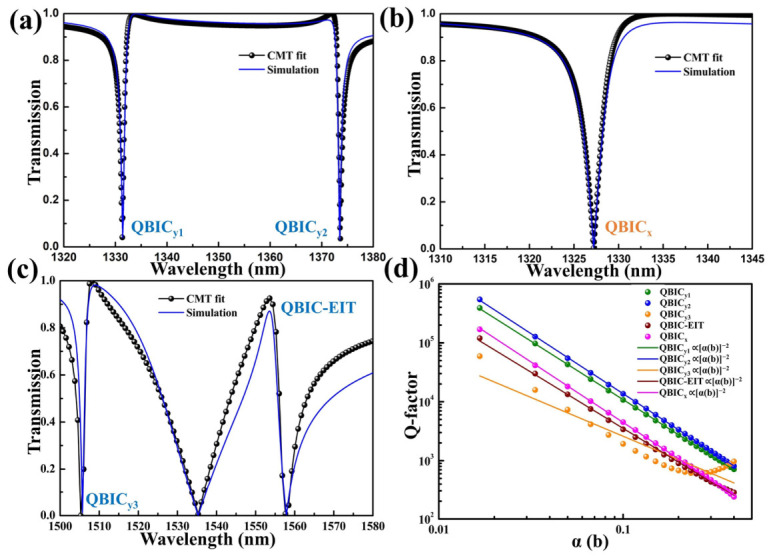
CMT analysis of QBIC and QBIC-EIT modes: (**a**) QBIC_y1_ and QBIC_y2_ mode comparisons between CMT fitting curves (dotted black lines) and simulated transmission spectra (solid blue lines). (**b**) Equivalent validation for QBIC_x_ mode, demonstrating agreement between theoretical fits and computational results. (**c**) Concurrent analysis for QBIC_y3_ and QBIC-EIT modes, highlighting modal coupling dynamics. (**d**) Q-factor evolution across resonant modes as a function of asymmetry parameter, *a*(b), illustrating divergent behavior at minimal offsets.

To model the SP-BIC resonance and its coupling with the SLM, we employ coupled mode theory (CMT). Within this framework, the transmission spectrum T(*ω*) is expressed as a function of angular frequency *ω* [62,63,64]:

(1) $$T(\omega )=\frac{t^{2}{\left(\omega -\omega_{0}\right)}^{2}+t^{2}\cdot \gamma^{2}+2\cdot r\cdot t(\omega -\omega_{0})\cdot \gamma }{{\left(\omega -\omega_{0}\right)}^{2}+\gamma^{2}}$$

where γ is the total radiative attenuation rate, and *ω*_0_ is the frequency at the resonance peak, which can be obtained by calculating the eigenfrequency of the metasurface [18]. Here, *t* and *r* represent the transmission and reflection coefficients of the reference substrate spectra, respectively, obtained through electromagnetic simulation of the bare substrate structure [65,66]. We provide a detailed introduction to the derivation process of Equation (S1) in Supplementary Material Section S1. As shown in Figure 3a,b, our CMT fitting for all quasi-SP-BIC resonances demonstrates excellent agreement with COMSOL simulation results. Furthermore, we model the interaction between the low-Q SLM mode and the high-Q BIC mode, mirroring the EIT effect in three-level atomic systems. The transparency window originates from SLM-QBIC mode coupling during QBIC-EIT resonance. For our CMT analysis, we simplify the coupling equations by excluding intermodal interactions. As shown in Figure 3c, the fitted transmission spectrum demonstrates close agreement with COMSOL simulations, with minor deviations attributable to neglected mode couplings [1,67].

**Figure 4:**
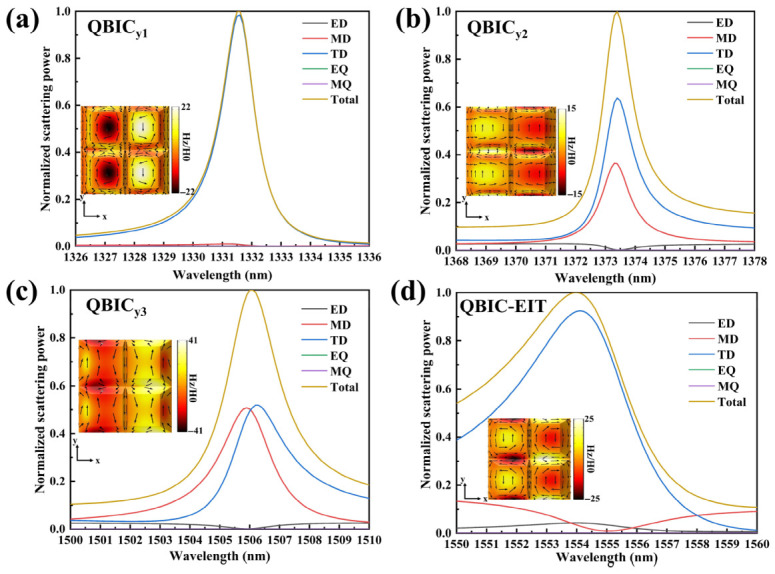
The multipole decomposition analysis of the four resonant modes under y-polarized illumination, plotting normalized far-field scattering intensities: (**a**) The QBIC_y1_ mode is dominated by the TD. (**b**) QBIC_y2_ features joint contributions from the TD and MD. (**c**) QBIC_y3_ similarly exhibits co-dominant TD and MD characteristics. (**d**) The QBIC-EIT mode displays predominant TD excitation. Insets show the corresponding magnetic field distributions, with black arrows indicating surface current density vectors.

Using the CMT, we extract the Q-factors of individual modes through the relationship *Q =* ω_0_*/2*γ [68,69], where ω_0_ is the resonance frequency (real part of the complex eigenfrequency) and γ is the leakage rate (imaginary part of the eigenfrequency). An appealing feature of quasi-BIC metasurfaces is their capacity to tailor the Q-factor and radiation properties through structural parameter tuning. We define the asymmetry parameter associated with the pyramid displacement as *a(b) = b/b*_1_, where *b*_1_ denotes the slit spacing between pyramids under perfect symmetry. The Q-factors of the respective modes are plotted against the asymmetry parameter *a*(*b*) in Figure 4d. Evidently, the Q-factor is proportional to the inverse square of the asymmetry parameter, *a*(*b*). As b decreases, all Q-factors increase monotonically, diverging to infinity as *b* → 0. This asymptotic behavior confirms all five modes as symmetry-protected SP-BICs [36]. The dependence of the Q-factor of the QBIC_y3_ mode on the asymmetry parameter deviates from the simple inverse-square law. This is mainly because a QBIC mode typically manifests as the scattering enhancement of a single dipole accompanied by the suppression of other dipoles in the multipole decomposition [70]. In contrast, the multipole decomposition of the QBIC_y3_ mode reveals a hybrid of TD and MD. The coexistence and interaction of different multipole moments affect the overall radiation rate. A detailed multipole analysis of the QBICs will be presented in the next section. A smaller asymmetry parameter yields a higher Q-factor. The resulting higher Q-factor leads to a narrower resonance linewidth, which, according to the standard definition of the FOM, directly enhances the sensor’s performance. Furthermore, we analyze the eigenfrequency components of all modes versus the offset parameter *b*. As shown in Figure S1, the total radiative attenuation rate, as presented with the red dotted line, increases with *b*, consistent with Q-factor calculations, while the eigenfrequency real component, as presented with the black dotted line, remains virtually unaffected by *b*. Resonance positions exhibit minimal shift with varying *b*, confirming exceptional spectral stability in the metasurface design despite fabrication tolerances. Furthermore, we introduced rounded corners and sidewall roughness to the metasurface unit cells and compared the Q-factors of all resonant modes before and after the roughness treatment, as shown in Figure S2. The results demonstrate that, despite the introduced roughness, most QBIC resonances and QBIC-EIT modes maintain their Q-factors without significant degradation, except for the QBIC_x_ resonance, which shows a relatively noticeable reduction. This indicates that these QBIC resonances and QBIC-EIT modes possess relatively stable performance against fabrication imperfections. The resonance quality of high-Q structures can be affected by the finite linewidth of the excitation source. One method to evaluate the structure’s response to a beam of finite width is to examine its angular dependence [49]. We calculated the transmission spectra of QBIC_x_, QBIC_y1_, and QBIC_y2_ under incident angles ranging from 0° to 2°, as shown in Figure S3 in the Supplementary Material. All these QBIC resonances exhibit a redshift that increases with the incident angle. Nevertheless, the resonances do not vanish, and their linewidths remain stable, indicating that the designed structure exhibits a certain robustness to angular deviations. Since the numerical aperture (NA) of a probe beam directly defines its angular spread (NA = *n*sin*θ*_max_), the demonstrated robustness of the QBIC resonances over a 0–2° incident angle range (corresponding to NA ≈ 0.035 in air) confirms that the platform is resilient to the typical angular divergence encountered in practical implementations with moderate-NA probe beams.

### 4.2. Multipole Expansion and Electromagnetic Field Analysis of Resonant Modes

To further elucidate this EIT-like and QBIC dual-resonance system, we simulated the electric and magnetic field profiles and performed a Cartesian multipole decomposition of the far-field scattering cross section near the resonance wavelengths [71,72]. The detailed methodology for multipole decomposition is provided in Supplementary Material Section S4. Figure 4 presents the multipole expansion analysis for the four resonances under y-polarized illumination, with scattering radiation intensities normalized for comparison. As clearly shown, these modes are predominantly governed by TD and magnetic dipole (MD) contributions. The insets in Figure 4 depict the magnetic field z-components and corresponding surface current distributions for each mode. Specifically, the QBIC_y1_ mode in Figure 4a exhibits concentrated magnetic field z-components at the pyramid centers, displaying dipolar patterns symmetric along the *y*-axis. Surface currents form circulating loops on both the top surfaces and sidewalls of each pyramid. Crucially, coupled circulating currents among the four pyramids collaboratively form a TD moment—a characteristic TD signature consistent with the multipole decomposition results.

Similarly, the QBIC_y2_ mode in Figure 4b also arises from the TD excitation. Its maximum magnetic field z-component localizes on the pyramid sidewalls oriented along the *x*-axis. Interconnected circulating currents between adjacent sidewalls establish a clear TD configuration. Concurrently, weaker magnetic field components at the pyramid centers, accompanied by sparse *x*-axis-oriented circulating currents, suggest reduced MD activity. It is interesting to note that the QBIC_x_ mode also exhibits strong TD character (see Figure S4 in the Supplementary Material). Here, the magnetic field z-component primarily concentrates on the pyramids’ upper surfaces, with surface currents forming circulating loops. However, although both the QBIC_y1_ and QBIC_x_ modes in the same spectral band are dominated by the TD response, their magnetic field distributions exhibit distinct characteristics due to different incident polarizations: the bright and dark mode distributions of QBIC_y1_ are symmetric about the *y*-axis, while those of QBIC_x_ are symmetric about the *x*-axis, directly corresponding to their respective excitation polarizations. This indicates that the polarization dependence induced by the geometric asymmetry of the structure also influences the polarization behavior of the TD.

In contrast, the QBIC_y3_ mode arises from the combined action of MD and TD components. The inset in Figure 4c reveals an elliptical distribution of the magnetic field z-component concentrated on x-oriented sidewalls, resembling the QBIC_y2_ mode. Here, sidewall current coupling generates toroidal moments. Distinctively, this mode simultaneously exhibits enhanced magnetic field components and corresponding circulating currents on y-oriented sidewalls, manifesting concurrent MD characteristics. This combination mechanism, quantitatively verified by multipole decomposition, shows close agreement with the observed electromagnetic field distributions.

We perform the multipole analysis of the QBIC-EIT hybrid mode, which reveals distinct operational regimes. The SLM mode near 1540 nm in Figure S5 of the Supplementary Material shows predominant MD, TD, and electric dipole (ED) contributions. Notably, despite symmetry breaking, its far-field scattering maintains displacement-independent flat spectral responses rather than resonant behavior. Concurrently, the QBIC resonance at 1554 nm, as shown in Figure 4d, demonstrates TD-dominated characteristics, namely toroidally coupled current loops bridging pyramid tops and *y*-axis sidewalls generate collective TD moments, while z-component magnetic field concentrations at geometric centers and sidewall junctions validate the toroidal excitation mechanism through multipole decomposition consistency. When symmetry is broken, the TD mode becomes dominant and suppresses the far-field scattering of other dipoles. The BIC mode near the SLM mode undergoes radiative leakage and transforms into a QBIC, resulting in an asymmetric Fano resonance peak in the transmission spectrum. As the asymmetry parameter b gradually increases, the SLM undergoes a frequency shift and approaches the QBIC located at 1554 nm, coupling with it to form a new QBIC-EIT mode.

### 4.3. Enhanced RI Sensitivity with the Asymmetric Metasurface

**Figure 5:**
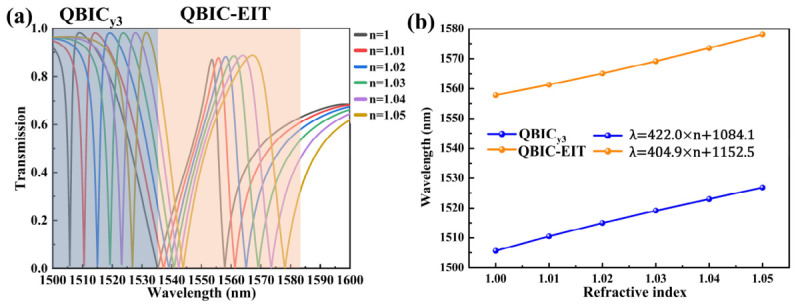
RI sensing of QBIC_y3_ and QBIC-EIT modes: (**a**) Evolution of transmission spectra for the QBIC_y3_ mode (blue-highlighted window) and QBIC-EIT resonance (orange-highlighted window) under varying ambient refractive indices. (**b**) QBIC_y3_ dip shift tracking (blue dotted-line markers) and QBIC-EIT peak shift monitoring (orange dotted-line markers).

The EIT-like window and QBIC resonance offer exceptional potential for high-sensitivity RI sensing due to their sharp, high-Q resonance. High-Q resonance implies that the field is largely confined within the structure, resulting in minimal outward radiative scattering loss. This enables a narrower linewidth, which in turn improves the achievable detection limit and FOM [49]. Despite this internal confinement, the evanescent tail extends 80–150 nm into the analyte (the 1/*e* penetration depth), ensuring practical accessibility for surface-bound molecules. Figure 5 demonstrates the excellent sensitivity of our dual-resonance metasurface design. The refractive index variation range for most gases falls within 1.0–1.1, whereas the refractive index range for most liquids lies between 1.3 and 1.5 [49]. To evaluate its sensing capability, we placed the metasurface in a 3 μm high gas cell during simulations and varied the background refractive index n from 1.00 to 1.05 in increments of 0.01 to emulate a gaseous environment. Under y-polarized illumination with offset b = 100 nm, even minute changes in RI induce significant redshifts in both the SP-BIC_y3_ resonance and EIT-like window positions, as shown in Figure 5a. To quantify the metasurface’s sensing capability, we define sensitivity S as the spectral shift per RI unit (RIU), i.e., S = Δλ/Δn. Figure 5b shows the linear fitting curves for the QBIC_y3_ and QBIC-EIT resonance. The sensitivities are S_QBICy3_ = 422.0 nm/RIU and S_QBIC-EIT_ = 404.9 nm/RIU. Another key sensing metric is FOM, defined as FOM = S/FWHM, where FWHM is the full width at half maximum of the transmission peak. This definition is rigorously valid for Lorentzian resonances. However, for EIT-like or Fano profiles that exhibit asymmetry, the FWHM still provides a practically useful measure of spectral sharpness and resolving power, albeit as an approximation. The widespread use of this metric in EIT, QBIC, and Fano resonance sensing literature [18,73] enables consistent cross-platform comparison, and we adopt it here for that purpose. When precise line-shape fitting is required, an alternative metric such as FOM* = S/FWHM_eff_ based on an asymmetric line-shape model could be employed, but this lies beyond the scope of the present work. The QBIC_y3_ resonance (FWHM = 1 nm) yields FOM = 422.0 RIU^−1^. The wider QBIC-EIT window (FWHM = 2.5 nm) achieves FOM = 162 RIU^−1^. We also investigated the QBIC_y1_ and QBIC_y2_ modes, as shown in Figure S6 in the Supplementary Material. These two resonant modes exhibit relatively lower sensitivity to environmental refractive index changes, with QBIC_y1_ demonstrating a sensitivity of S = 866.0 nm/RIU and QBIC_y2_ achieving S = 218.9 nm/RIU. Despite its lower sensitivity, QBIC_y2_’s exceptionally narrow linewidth (FWHM = 0.8 nm) results in a higher FOM.

**Figure 6:**
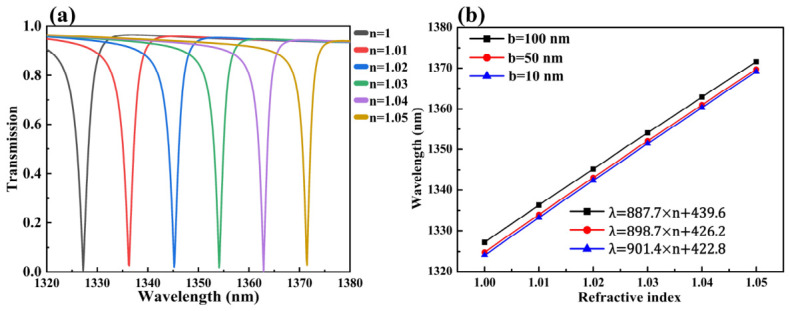
Refractometric sensing and displacement-dependent sensitivity analysis of the QBIC_x_ Mode: (**a**) Evolution of the QBIC_x_ transmission spectra with surrounding RI. (**b**) Sensitivity calibration plots for QBIC_x_ at different displacement parameters b, showing linear regression fits of resonance wavelength versus RI.

Additionally, the QBIC_x_ resonance induced in the 1300 nm band under x-polarized illumination exhibits ultra-high RI sensing sensitivity. As shown in Figure 6a, the QBIC_x_ resonance undergoes significant redshifting with minimal changes in RI (Δn = 0.1). Its linear fitting curve in Figure 6b yields an RI sensitivity of S = 887.7 nm/RIU, with FWHM = 1 nm and FOM = 887.7 RIU^−1^. Intriguingly, the sensitivity remains largely unaffected as structural offset b decreases. With diminishing b, the metasurface’s symmetry breaking becomes increasingly subtle, causing the QBIC’s Q-factor to approach the theoretically infinite Q of a true BIC. This corresponds to a reduced radiative lifetime and a narrower resonance linewidth. At b = 50 nm, FWHM = 0.6 nm, and at b = 10 nm, FWHM = 0.1 nm. While reducing b does not enhance RI sensitivity directly, the metasurface’s sensing FOM improves dramatically due to the sharp decrease in linewidth. Specifically, FOM reaches 1497.3 RIU^−1^ at b = 50 nm and 9014 RIU^−1^ at b = 10 nm. This designed metasurface enables dual-band, multi-resonance RI sensing with exceptional sensitivity and FOM across polarizations, demonstrating strong potential as a highly efficient RI sensor.

Our designed metasurface structure demonstrates applicability for sensing in high-refractive-index liquid environments. In our simulations, a 500 nm thick analyte layer was applied to the metasurface, with the analyte’s refractive index varied from 1.3 to 1.5 in steps of 0.05. The corresponding sensing performance of the QBIC_y1_, QBIC_y2_, and QBIC_x_ modes is shown in Figure S7. In high-refractive-index environments, the refractive index sensitivity of QBIC_x_, QBIC_y1_, and QBIC_y2_ is considerably lower than that in low-refractive-index gas environments. This reduction occurs because the high-refractive-index liquid increases the asymmetry in the dielectric environment, thereby broadening the resonance linewidth and consequently degrading the sensing performance [74]. Furthermore, the existing literature also indicates that the sensing performance of all-dielectric metasurfaces in high-refractive-index liquids is generally inferior to their performance in low-refractive-index gas detection [75]. Our results align with this established trend. The calculated sensitivities are 190 nm/RIU for QBIC_y1_, 135 nm/RIU for QBIC_y2_, and 138.2 nm/RIU for QBIC_x_. Although these values are lower than those in gaseous environments due to reduced field confinement contrast, they remain competitive. For instance, our sensitivities are still higher than those reported for a silicon-based superstructure operating in the same detection wavelength band [76].

Furthermore, as shown in Figure S7d, we analyzed the effect of analyte thickness on the metasurface’s sensing performance. When the analyte thickness was increased from 250 nm to 1000 nm, the refractive index sensitivity exhibited only minimal variation. This indicates that the analyte thickness has an insignificant impact on the refractive index sensitivity, highlighting a favorable characteristic for practical device fabrication and measurement scenarios. Previous studies suggest that incorporating two-dimensional materials (such as perovskites) onto the metasurface could enhance aqueous solution sensing performance due to their excellent stability and water molecule adsorption properties [77]. In summary, although the refractive index sensing performance is reduced in high-refractive-index environments, our structure remains suitable for sensing applications under such conditions.

We compared the sensing performance of our designed device with recent metasurface-based sensors in the infrared regime, as summarized in Table 1. It is worth noting that conventional single-resonance sensing may suffer from inaccuracy and unreliability due to spectral shifts induced by environmental fluctuations in temperature, humidity, and intrinsic instrumental drift. This limitation can be effectively mitigated by transitioning from single- to dual-resonance sensing strategies, where the dependence of resonance wavelengths on the ambient refractive index can be well described by first-order perturbation theory [78]. A broader comparison with representative metasurface sensors is provided. The anapole-mode-based Si elliptical metasurface [79] supports only a single resonance and exhibits lower sensitivity and FOM in gaseous environments than our platform. The all-dielectric a-Si dual-resonance metasurface [76] shows high sensitivity in liquid environments but has an FOM of only 66, which is substantially lower than that of our work. Conversely, a dual-resonance gold plasmonic metasurface [80] achieves a narrower FWHM in high-refractive-index environments, resulting in a relatively high FOM; however, its refractive index sensitivity is lower than that achieved in our platform. Polarization-selective quasi-BIC resonances have been reported in other all-dielectric metasurfaces [81]. In comparison, our LN-based tilted pyramid platform simultaneously supports three independent high-Q resonances with polarization control and exhibits resonance robustness against asymmetry variations—advantages not demonstrated in [81]. While most prior works have focused on either single- or dual-resonance schemes, our metasurface supports multiple resonances across different wavelength bands and exhibits strong polarization dependence, offering distinct advantages for broadband detection. Although the FOM of our sensor is moderate compared to other works, the maximum sensitivity of 887.7 nm/RIU achieved here surpasses that of many recently reported metasurface sensors. Such high sensitivity is particularly advantageous in gas sensing applications, where minute refractive index changes need to be detected.

## 5. Conclusions

In summary, we theoretically designed a dielectric metasurface that supports multiple SP-BICs and a QBIC-EIT hybrid mode. Enabled by a periodic array of displaced LN pyramids, the structure exhibits distinct resonant behaviors under different polarization states and across separate wavelength bands. Through systematic analysis using CMT and multipole decomposition, we clarified that these resonances originate from either pure TD excitations or hybridized TD–MD interactions. The proposed metasurface demonstrates exceptional RI sensing performance, with sensitivities as high as 887.7 nm/RIU and 866.0 nm/RIU for the QBIC_x_ and QBIC_y1_ modes, respectively, and 422.0 nm/RIU and 404.9 nm/RIU for the QBIC_y3_ and QBIC-EIT modes. Importantly, the resonance frequencies show minimal dependence on the geometric displacement parameter *b*, ensuring strong fabrication tolerance—significant structural deviations introduce only negligible spectral shifts and no appreciable degradation in sensitivity. This work not only advances the practical implementation of high-performance refractive index sensing metasurfaces but also enables multi-band, high-sensitivity detection, offering a novel technical pathway for applications such as gas refractive index monitoring.

## Figures and Tables

**Table 1:** Performance comparison of refractive index sensors in the near-infrared region.

Year	Structure	Resonance Mode	Maximum Sensitivity (nm/RIU)	FOM	Polarization Dependence	Resonance Number	Detection Range
This work	LN pyramid	QBIC&EIT-like	887.7	887.7	yes	5	1–1.05 1.3–1.5
2025 [73]	Dielectric Dumbbell hole	QBIC&anapole	745	1650	yes	2	1–1.04 1.3–1.5
2023 [78]	Si cylindroid	QBIC	122.2	206.1	no	2	1–1.5
2023 [80]	Gold plasma film	SPP-SLR	514	1431	no	2	1–1.384
2025 [76]	Amorphous Si column	QBIC	413	66	no	2	1.3–1.6
2025 [79]	Si cylindroid with hole	anapole	393.4	596.1	no	1	1–1.05
2026 [81]	GaP bow-tie-shaped nanoholes	QBIC	342	217.1	yes	4	1.3–1.4

## Data Availability

The data presented in this study are available on request from the corresponding authors.

## Supplementary Materials

The following supporting information can be downloaded at: https://www.mdpi.com/article/10.3390/s26092632/s1, Section S1: Coupled-mode theory for QBICs and QBIC-EIT; Table S1: Fitting parameters for the five modes in CMT analysis; Figure S1: Real and imaginary parts of the eigenfrequencies for each mode as a function of the offset parameter b; Section S2: Q-factor degradation analysis due to fabrication roughness; Figure S2: Q-factor degradation analysis due to fabrication roughness; Figure S3: Analysis of QBIC Mode Stability under Angular Variation; Section S3: Multipole expansion and scatting power; Figure S4: Multipole decomposition of the QBIC_x_ mode; Figure S5: Multipole decomposition of the SLM mode; Section S4: Further Analysis on the Sensitivity of Refractive Index Sensing; Figure S6: The refractive index sensing of QBIC_y1_ and QBIC_y2_; Figure S7. Liquid high-refractive-index sensing and resonance-wavelength-based sensitivity analysis of QBIC_y1_, QBIC_y2_, and QBIC_x_ modes.

## Abbreviations

The following abbreviations are used in this manuscript:

BIC	Bound states in the continuum
EIT	Electromagnetically induced transparency
RI	Refractive index
S	Sensitivity
FOM	Figure of merit
