# Supplementary material for: Enhanced Sensing Enabled by Multi-Resonant QBIC-EIT and SP-BIC in Pyramidal LiNbO3 Metasurfaces
Source: Sensors (Basel). 2026 Apr 24;26(9):2632. doi: 10.3390/s26092632 (PMC13165597; doi:10.3390/s26092632)
Supplement: Supplementary file 1 [file sensors-26-02632-s001.zip › sensors-4237975-supplementary.pdf]

SUPPLEMENT MATERIAL:

Polarization-Dependent Dual-Resonances for High-Sensitivity Refractive Index Sensing

S1. Coupled-mode theory for QBICs and QBIC-EIT

By applying coupled-mode theory (CMT), the dynamic equations for the amplitude a of the resonance mode can by written as[1-4]:

$$\frac{dA}{dt} = (j\omega_0 - \gamma)A + \kappa S_+ \tag{S1}$$

$$S_- = CS_- + a\kappa \tag{S2}$$

Here, A represents the resonant amplitude, ω_0 is the resonant frequency, and γ represent the total radiation losses. S_+ and S_- denote the input and output waves from port 1, respectively. The scattering matrix expressed as:

$$C = \exp(j\phi)\begin{pmatrix} r & jt \\ jt & r \end{pmatrix} \tag{S3}$$

Describes the background reflectance and transmission without the resonance, where r, t and ϕ are real constants with $r^2 + t^2 = 1$ [5]. The vector $\kappa = \begin{bmatrix} d_1 & d_2 \end{bmatrix}^T$ represents the coupling coefficient between the outgoing waves at the ports and the resonant mode. Due to time-reversal symmetry and energy conservation, d needs to satisfy the following equation:

$$\langle d \mid d \rangle = 2\gamma \tag{S4}$$

$$C|d\rangle^* = -|d\rangle \tag{S5}$$

Using Eq. S4 and Eq. S5, we can determine d_1 and d_2, and consequently the scattering matrix S for overall system as:

$$S = \exp(j\phi)\left\{\begin{bmatrix} r & jt \\ jt & r \end{bmatrix} + \frac{\gamma}{j(\omega - \omega_o) + \gamma}\begin{bmatrix} -(r \pm jt) & \mp(r \pm jt) \\ \mp(r \pm jt) & -(r \pm jt) \end{bmatrix}\right\} \tag{S6}$$

Here the $\pm$ sign corresponds to the case where the resonant mode is even(odd) with respect to the mirror plane, in which case $d_1 = +(-)d_2$. From Eq. S6, the transmission T can be derived as:

$$T(\omega) = \frac{t^2(\omega - \omega_0)^2 + t^2 \cdot \gamma^2 \mp 2 \cdot r \cdot t(\omega - \omega_0) \cdot \gamma}{(\omega - \omega_0)^2 + \gamma^2} \tag{S7}$$

The fitting parameters for the five modes in CMT analysis are listed in Table S1. To simplify the fitting equations, the coupling between different modes was neglected when fitting the data in Fig. 3(c). This approximation resulted in discrepancies between the CMT-fitted results and the transmission curves obtained from COMSOL simulations, although the peak profiles remain consistent.

Table S1 Fitting parameters for the five modes in CMT analysis.

Mode	ω_0 (THz)	γ(THz)
$QBIC_{y1}$	225.30	0.112
$QBIC_{y2}$	218.44	0.093
$QBIC_x$	226.10	0.304
$QBIC_{y3}$	199.18	0.141
QBIC-EIT	192.80	0.262
SLM	195.40	2.293

Combining CMT and eigenfrequency analysis, we calculated the variation of Q-factors for each mode as a function of the offset parameter b. The real and imaginary parts of the eigenfrequencies for each mode, plotted against b, are shown in Figure. S1. The imaginary part of the eigenfrequencies increases with b, which aligns with the Q-factor trends calculated in Fig. 3(d). In contrast, the real part of the eigenfrequencies remains nearly constant as b varies, indicating that the designed metasurface exhibits robust stability against changes in b, and the resonance peaks are minimally affected by variations in the offset parameter.

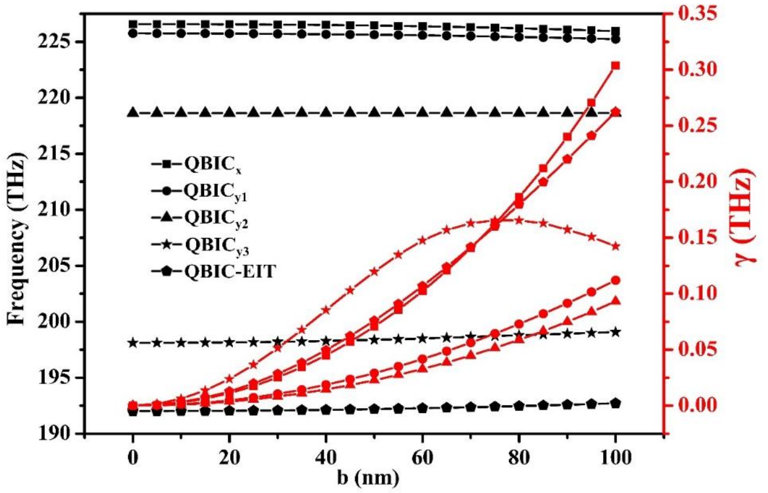

Figure. S1. Real (black dotted lines) and imaginary (red dotted lines) parts of the eigenfrequencies for each mode as a function of the offset parameter b.

S2. Q-factor degradation analysis due to fabrication roughness

We introduce rounded corners and sidewall roughness to the metasurface unit cells and compared the Q-factors of all resonant modes before and after the roughness treatment, as shown in Figure. S2. To prevent overlapping of multiple modes from obscuring the curves in the plot, we have split Figure. S2 into two panels: (a) compares the Q-factors of $QBIC_{y1}$, $QBIC_{y2}$, and $QBIC_x$ with and without roughness, while (b) shows the corresponding comparison for $QBIC_{y3}$ and the QBIC-EIT mode. In the figure, data points represent the relationship between the Q-factor of ideal (unroughened) QBIC resonances and the asymmetry parameter, while solid lines depict the corresponding trends after the introduction of roughness. It is evident that, aside from a noticeable reduction in the Q-factor of the $QBIC_x$ mode, all other modes exhibit no significant degradation after the roughness treatment. The results demonstrate that despite the introduced roughness, most QBIC resonances and QBIC-EIT modes maintain their Q-factors without significant degradation, except for the $QBIC_x$ resonance which shows a relatively

noticeable reduction. This indicates that these QBIC resonances and QBIC-EIT modes possess relatively stable performance against fabrication imperfections.

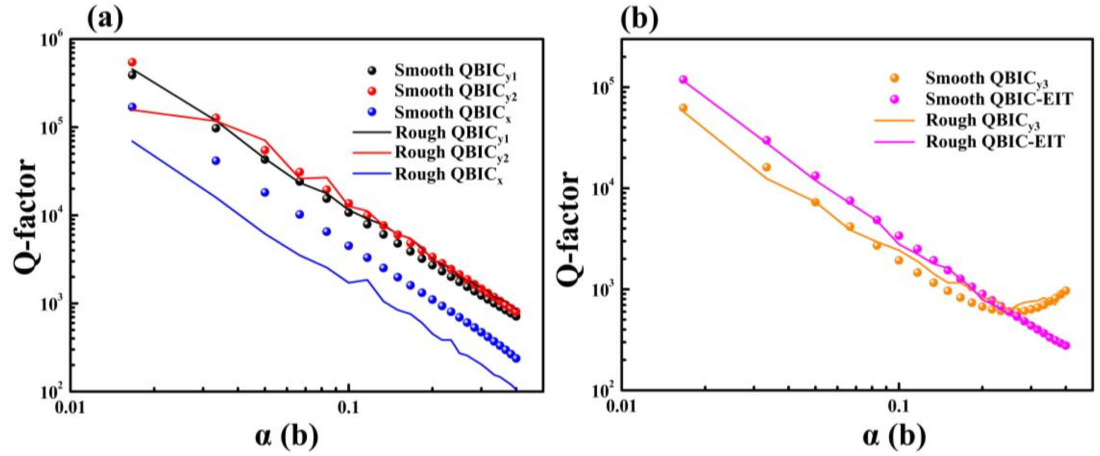

Figure. S2. (a) compares the Q-factors of $QBIC_{y1}$, $QBIC_{y2}$, and $QBIC_x$ with and without roughness, while (b) shows the corresponding comparison for $QBIC_{y3}$ and the QBIC-EIT mode.

S3. Analysis of QBIC mode stability under angular variation

The transmission spectra of $QBIC_x$, $QBIC_{y1}$, and $QBIC_{y2}$ under incident angles ranging from 0° to 2° are shown in Figure S3. All these QBIC resonances exhibit either redshift or blueshift with increasing incident angle. The $QBIC_x$ and $QBIC_{y1}$ modes demonstrate minimal redshift, while the $QBIC_{y2}$ mode shows a pronounced blueshift as the angle increases. This distinct behavior of $QBIC_{y2}$ may be attributed to its opposite asymmetry in spectral line shape compared to the $QBIC_{y1}$ mode. Nevertheless, all resonances remain stable without vanishing, and their linewidths are well preserved, indicating that the designed structure possesses considerable robustness against angular deviations.

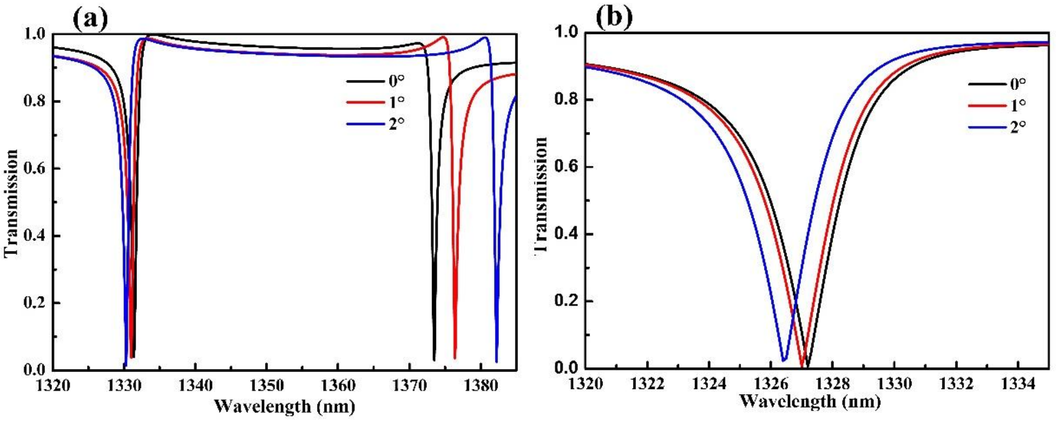

Figure S3. (a) $QBIC_{y1}$ and $QBIC_{y2}$ transmission spectra (0–2° incidence). (b) $QBIC_x$ transmission spectra (0–2° incidence).

S4. Multipole expansion and scatting power

We compute the total scattering power σ spectra of the cartesian multipoles in free space as[6-8]:

$$\sigma = \frac{2\omega^4}{3c^3}|P|^2 + \frac{2\omega^4}{3c^3}|M|^2 + \frac{2\omega^5}{3c^4}(P \cdot T) + \frac{2\omega^6}{3c^5}|T|^2 + \frac{\omega^6}{5c^5}Q_{\alpha\beta}Q_{\alpha\beta} + \frac{\omega^6}{20c^5}M_{\alpha\beta}M_{\alpha\beta} + \frac{2\omega^6}{15c^5}(M \cdot \langle R_M^2 \rangle + o(\frac{1}{c^5}) \tag{S8}$$

where P represent electric dipole moment; M represent magnetic dipole moment; T represent toroidal dipole moment; $Q_{\alpha\beta}$ represent electric quadrupole moment; $M_{\alpha\beta}$ represent magnetic quadrupole moment; $\langle R_M^2 \rangle$ represent mean-square radius of magnetic dipole distribution. These multipole components can be derived from Eq. S9:

$$\begin{aligned} P &= \frac{1}{i\omega}\int j d^3r; \\ M &= \frac{1}{2c}\int (r \times j) d^3r; \\ T &= \frac{1}{10c}\int [(r \cdot j)r - 2r^2 j] d^3r; \\ Q_{\alpha\beta} &= \frac{1}{i\omega}\int [r_\alpha j_\beta + r_\beta j_\alpha - \frac{2}{3}(r \cdot j)] d^3r; \\ M_{\alpha\beta} &= \frac{1}{3c}\int [(r \times j)_\alpha r_\beta + (r \times j)_\beta r_\alpha] d^3r; \\ \langle R_M^2 \rangle &= \frac{1}{2c}\int (r \times j) r^2 d^3r; \end{aligned} \tag{S9}$$

Figure S4 illustrates the multipole decomposition of the $QBIC_x$ mode. The $QBIC_x$ mode is primarily dominated by the toroidal dipole (TD), as illustrated in the inset. The magnetic field's z-component is localized at the pyramid tips, forming a vortex-like circulation. Surface currents between the four pyramids interact to generate a toroidal dipole, as evidenced by their coupled flow patterns. Figure S5 demonstrates the multipole decomposition of the SLM mode under symmetric and asymmetric conditions. In both cases, the scattering intensity of the SLM mode originates from a mixture of TD, magnetic dipole (MD), and electric dipole (ED) contributions. Notably, no resonant peaks are observed in either configuration.

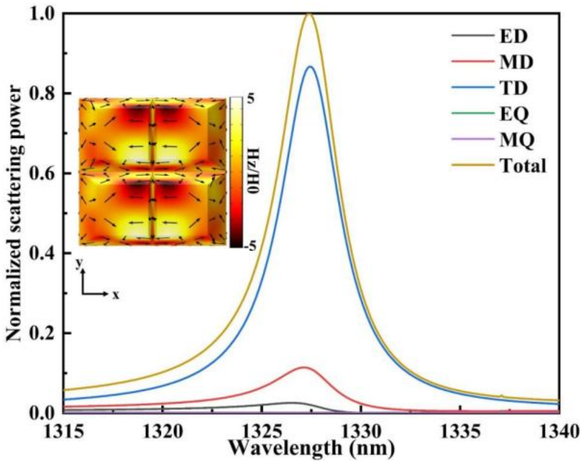

Figure. S4. Multipole decomposition of the $QBIC_x$ mode.

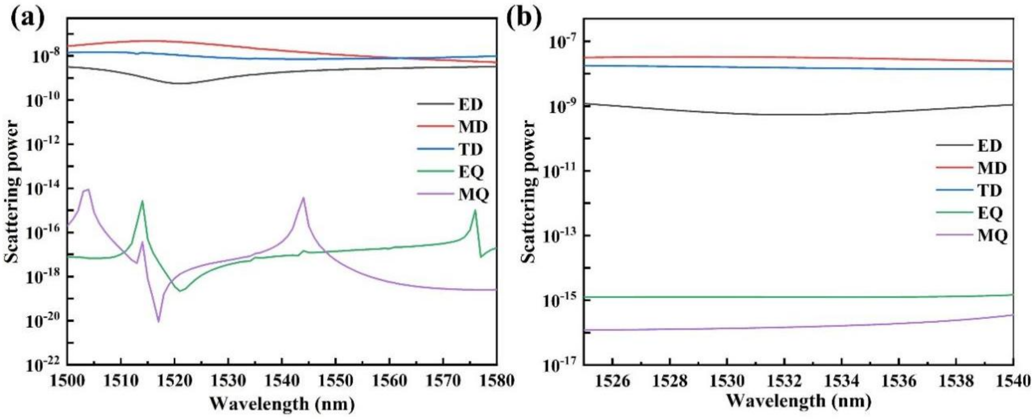

Figure. S5. Multipole decomposition of the SLM mode under (a) symmetric and (b) asymmetric conditions.

S5. Further Analysis on the Sensitivity of Refractive Index Sensing

Figure S6 shows the refractive index sensing transmission curves and sensitivity fitting curves for $QBIC_{y1}$ and $QBIC_{y2}$. The refractive index sensing sensitivities of $QBIC_{y1}$ and $QBIC_{y2}$ are 866.0 nm/RIU and 218.9 nm/RIU, respectively. Given that both modes exhibit a full width at half maximum (FWHM) of 0.8 nm, their figures of merit (FOMs) are 1082.5 RIU^{-1} and 273.6 RIU^{-1}. Figure S7 presents the refractive index sensing performance evaluation of $QBIC_{y1}$, $QBIC_{y2}$, and $QBIC_x$ in a high-refractive-index liquid environment. In our simulations, a 500-nm-thick analyte layer was applied to the metasurface, with the analyte's refractive index varied from 1.3 to 1.5 in steps of 0.05. The corresponding sensing performance of the $QBIC_{y1}$, $QBIC_{y2}$, and $QBIC_x$ modes is shown in Fig. S7. When a 500-nm-thick high-refractive-index liquid analyte is applied to the metasurface structure, the resonance linewidths of $QBIC_{y1}$, $QBIC_{y2}$, and $QBIC_x$ significantly broaden. This phenomenon can be attributed to symmetry breaking in the environmental refractive index distribution, which degrades the quality factor of the resonances, as previously reported in the literature[9]. The increased resonance linewidth reduces the field localization capability of the modes, leading to a notable decrease in the metasurface's sensing performance. The calculated sensitivity values are 190 nm/RIU for $QBIC_{y1}$, 135 nm/RIU for $QBIC_{y2}$, and 138.2 nm/RIU for $QBIC_x$. Furthermore, as shown in Figure. S7(d), we analyzed the effect of analyte thickness on the metasurface's sensing performance. When the analyte thickness was increased from 250 nm to 1000 nm, the refractive index sensitivity exhibited only minimal variation. This indicates that the analyte thickness has an insignificant impact on the refractive index sensitivity, highlighting a favorable characteristic for practical device fabrication and measurement scenarios.

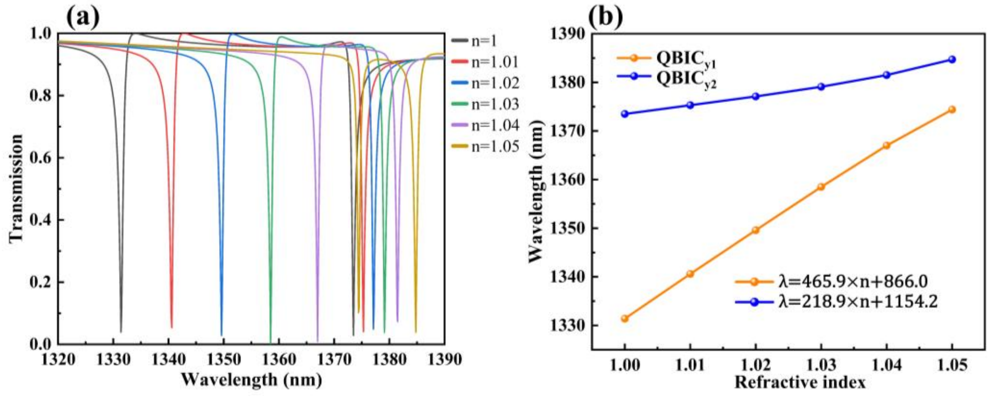

Figure. S6. Refractive index sensing transmission curves and sensitivity fitting curves for $QBIC_{y1}$ and $QBIC_{y2}$.

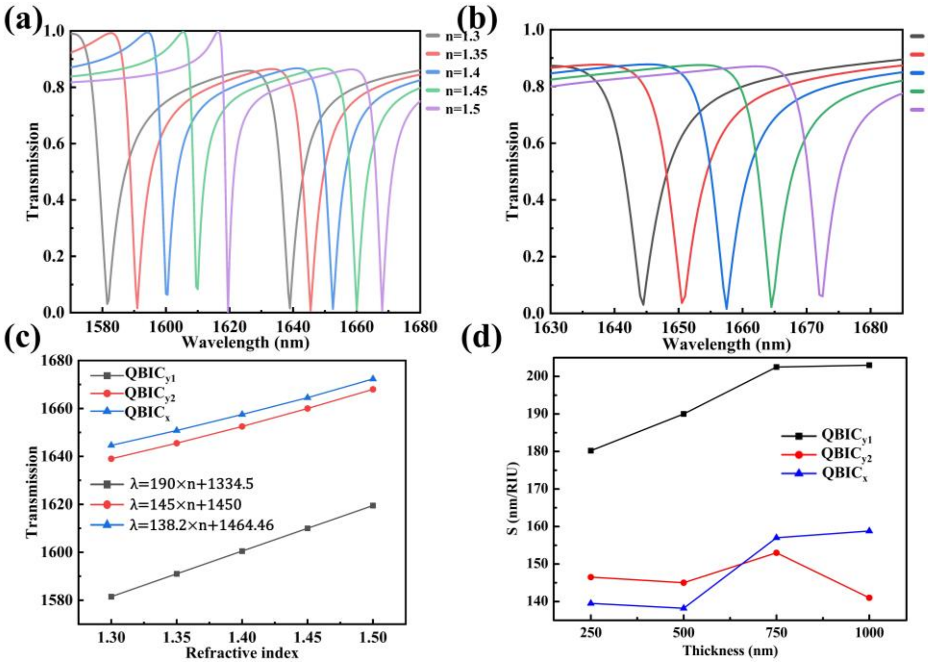

Figure. S7. Liquid high-refractive-index sensing and resonance-wavelength-based sensitivity analysis of $QBIC_{y1}$, $QBIC_{y2}$, and $QBIC_{x}$ modes. (a) Evolution of transmission spectra for $QBIC_{y1}$ and $QBIC_{y2}$ with varying ambient RI. (b) Evolution of $QBIC_{x}$ transmission spectra with varying ambient RI. (c) Resonance wavelength monitoring of $QBIC_{y1}$ (blue dotted line), $QBIC_{y2}$ (orange dotted line), and $QBIC_{x}$ (red dotted line) modes. (d) Effect of analyte thickness on $QBIC_{y1}$ (blue dotted line), $QBIC_{y2}$ (orange dotted line), and $QBIC_{x}$ (red dotted line) modes.

References:

1. Fan, S.; Suh, W.; Joannopoulos, J. D., Temporal coupled-mode theory for the Fano resonance in optical resonators. *Journal of the Optical Society of America. A, Optics, image science, and vision* **2003,** 20 (3), 569-72.
2. Xu, H. X.; Li, K.; Yang, L.; Qian, J. R., Temporal coupled-mode theory for resonators. In *Proceedings of 2011 Cross Strait Quad-Regional Radio Science and Wireless Technology Conference*, (2011); Vol. 1, pp 82-84.
3. Ruan, Z.; Fan, S., Temporal Coupled-Mode Theory for Fano Resonance in Light Scattering by a Single Obstacle. *The Journal of Physical Chemistry C* **2010,** 114, (16), 7324-7329.
4. Overvig, A.; Mann, S. A.; Alù, A., Spatio-temporal coupled mode theory for nonlocal metasurfaces. *Light: Science & Applications* **2024,** 13, (1), 28.
5. Fan, S.; Joannopoulos, J. D., Analysis of guided resonances in photonic crystal slabs. *Physical Review B* **2002,** 65, (23), 235112.
6. Kaelberer, T.; Fedotov, V. A.; Papasimakis, N.; Tsai, D. P.; Zheludev, N. I., Toroidal Dipolar Response in a Metamaterial. *Science* **2010,** 330, (6010), 1510-1512.
7. Basharin, A. A.; Kafesaki, M.; Economou, E. N.; Soukoulis, C. M.; Fedotov, V.

A.; Savinov, V.; Zheludev, N. I., Dielectric Metamaterials with Toroidal Dipolar Response. *Physical Review X* **2015,** 5, (1), 011036.
8. Alaee, R.; Rockstuhl, C.; Fernandez-Corbaton, I., Exact Multipolar Decompositions with Applications in Nanophotonics. *Advanced Optical Materials* **2019,** 7, (1), 1800783.
9. Hu, H.; Lu, W.; Antonov, A.; Berté, R.; Maier, S. A.; Tittl, A., Environmental permittivity-asymmetric BIC metasurfaces with electrical reconfigurability. *Nature Communications* **2024,** 15, (1), 7050.
